# Promising Fluorine-Free Ion Exchange Membranes Based on a Poly(ether-block-amide) Copolymer and Sulfonated Montmorillonite: Influence of Different Copolymer Segment Ratios

**DOI:** 10.3390/membranes14010017

**Published:** 2024-01-06

**Authors:** Manhal H. Ibrahim Al-Mashhadani, Khirdakhanim Salmanzade, András Tompos, Asmaa Selim

**Affiliations:** 1 Institute of Materials and Environmental Chemistry, HUN-REN Research Centre for Natural Sciences, Magyar Tudósok Körútja 2, H-1117 Budapest, Hungary; manhal.ibrahim@ilps.uobaghdad.edu.iq (M.H.I.A.-M.); ksalmanzade5@gmail.com (K.S.); asmaa.selim@ttk.hu (A.S.); 2 Hevesy György Doctoral School of Chemistry, Eötvös Loránd University, Pázmány Péter sétány 1/A, H-1117 Budapest, Hungary; 3 Institute of Laser for Postgraduate Studies, University of Baghdad, 10070 Baghdad, Iraq; 4 Chemical Engineering and Pilot Plat Department, Engineering and Renewable Energy Research Institute, National Research Centre, 33 El Bohouth Street, Giza 12622, Egypt

**Keywords:** cation exchange membrane, copolymer, fluorine-free, PEBAX, sulfonated montmorillonite, inorganic–organic hybrid, fuel cell, ion conductivity

## Abstract

Novel composite membranes employing a poly(ether-block-amide) (PEBAX) copolymer and sulfonated montmorillonite (S-MMT) as a filler were developed. The ratio of polyether to polyamide blocks was investigated using PEBAX 2533 and PEBAX 4533 based on the membrane properties and performance. Additionally, the effect of the changing filler ratio was monitored. The interaction between the S-MMT as nanofiller and the polymer matrix of PEBAX2533 and PEBAX4533 as well as the crystalline nature and thermal and mechanical stability of the composite membranes were evaluated using Fourier Transform Infrared Spectroscopy (FT-IR), X-ray diffraction (XRD), thermogravimetric analysis (TGA), and tensile test. The composite membrane with 7 wt.% S-MMT showed the highest water uptake of 21% and 16% and an acceptable swelling degree of 16% and 9% for PEBAX 2533 and PEBAX 4533 composite membranes, respectively. In terms of water uptake and ion exchange capacity at room temperature, the new un-protonated membranes are superior to un-protonated Nafion. Meanwhile, with the same S-MMT content, the ion conductivity of PEBAX 2533 and PEBAX 4533 composite membranes is 2 and 1.6 mS/cm, and their ion exchange capacity is 0.9 and 1.10 meq/g.

## 1. Introduction

The demand for energy, as well as people’s reliance on it, is growing at a rate that is proportional to both the rate of technological innovation and the rate of population growth. Fuel cells are promising methods for the conversion of energy that can satisfy this need for energy [1]. Proton exchange membrane fuel cells (PEMFCs) are attracting greater attention due to their favorable operating conditions, wide applications, and high efficiencies [2]. The proton exchange membrane (PEM) is sometimes referred to as the “heart” of a PEM fuel cell because of its key role in the fuel cell’s operation [3,4,5]. PEMFC requires that membranes have certain characteristics, including excellent proton conductivity, low fuel crossover, thermal and chemical stability, high mechanical strength, and a low cost [6]. Due to its high proton conductivity and outstanding mechanical strength, Nafion is the most widely used commercial membrane [7,8,9]. It is a perfluorosulfonic (PFSA) polymer. When the membrane is wet, the sulfonic acid groups as well as the phase separation between the hydrophilic groups and the hydrophobic PTFE backbone ensure that protons can easily pass through it. The PTFE backbone also provides high mechanical stability [10]. 

Poly(ether-block-amide) membranes, under the tradename of PEBAX^®^ [11,12], are advantageous because they have attractive properties including high permeability, and high selectivity and durability. The mechanical stability of the copolymer is controlled by the crystalline structure of the polyamide segments, while the amorphous structure of the polyether segments ensures the high permeability of organic substances [13,14]. The features are related to the polymer structure [15]. PEBAX has a rigid polyamide segment and a flexible polyether segment covalently bonded together to form a thermoplastic copolymer. PEBAX copolymers are synthesized through copolymerization of several forms of poly(amides) like PA6, PA66, and PA12 with different types of poly(ethers) like ethylene oxide and poly (tetra methylene oxide) [16]. The mechanical stability of the copolymer is controlled by the crystalline structure of the polyamide segments, while the amorphous structure of the polyether segments ensures a high permeability to organics [14]. Gas separation, pervaporation, ultrafiltration, and nanofiltration are some of the applications that have found success using PEBAX membranes [17]. PEBAX 2533 dominates the hydrophobic character due to the high percentage of poly(tetramethylene oxide) (PTMO) segments (80%) compared to 20% of a polyamide segment (PA12) [18]. Pervaporative removal of organic molecules (such as acetone, butanol, and ethanol) from aqueous solutions was accomplished using membranes based on PEBAX 2533 [19]. Amongst PEBAX polymers, it has the highest polyether content. PEBAX 4533, like PEBAX 2533, consists of a polyamide (PA12) hard segment and (PTMO) soft segment, but the polyether content is only 55% [20]. The combination of these two components in PEBAX is achieved using a co-polymerization procedure, leading to the development of a material that demonstrates an effective combination of the characteristics found in both polyamide and polyether [20]. The incorporation of PA12 and PTMO segments inside the PEBAX polymer matrix results in a highly adaptable material that exhibits desirable characteristics such as flexibility, toughness, and excellent resistance to chemical degradation. The amorphous PTMO regions are flexibly arranged, which allows PEBAX to absorb significant amounts of water and swell accordingly.

Layered silicates are considered effective inorganic particles that have been shown to contribute to reduce permeability when exfoliated and used as selectivity-improving fillers in a polymer matrix. Montmorillonite (MMT) as a layered silicate is often used as fillers in membranes to improve the separation efficiency. Nevertheless, the presence of hydroxyl end groups on the surface of MMT facilitates water absorption and subsequent swelling. These groups also pose a challenge in terms of effective mixing and surface interactions with large polymer molecules. Generally, MMT is modified prior to use to enhance its effectiveness in membrane applications. The modification process usually offers two distinct advantages. First, it facilitates the increase in the distance between the layers, which allows macromolecules to intercalate into the montmorillonite (MMT) structure. Second, the introduction of organic compounds that are linked to the layered silicates promotes the compatibility of MMT with the polymer matrix. Alkylammonium salts have often been used as a modifier of MMT. Nevertheless, the presence of these salts might result in the development of a hydrophobic nature and an enlarged interlayer gap in MMT. These factors can have a detrimental effect on the transport processes [21]. MMT has to be doped by properly selected hydrophilic modifiers to make it compatible with the hydrophilic polymer matrix. 

Taking into account the successful application of PEBAX and MMT in the membrane technology in many application areas, we used PEBAX polymers for the first time to prepare cation exchange membranes. Different ratios between polyether and polyamide segments (PEBAX 2533 and PEBAX 4533) were investigated based on the performance. Additionally, MMT was used as filler for the PEM membranes after sulfonation (S-MMT). The influence of a different S-MMT content on PEBAX, water uptake, swelling degree, ion exchange capacity, ion conductivity and crystallinity, and mechanical and thermal properties has been investigated. The present study is of significant importance as it pioneers the development of a fluorine-free ion exchange membrane with a unique blend of a PEBAX copolymer and sulfonated montmorillonite. Examining different ratios of copolymer segments on this membrane offers a breakthrough opportunity to improve ion exchange properties. By exploring this new combination, this research potentially addresses environmental concerns, offers a sustainable alternative to fluorine-based PEMs, and contributes to the development of fuel cell technologies toward more efficient and environmentally friendly solutions.

## 2. Experimental Methodology

### 2.1. Materials

PEBAX 2533 and PEBAX 4533 were kindly provided by Arkema (Colombes, France) for our collaborating partner. PEBAX 2533 consists of poly(tetramethylene oxide) (PTMO) and polyamide12 (PA12) (20 wt%) and for PEBAX 4533, it contains polyamide 12 (PA12) (∼50 wt%) and poly(tetramethylene oxide) (PTMO) segments. Na-montmorillonite (MMT) was supplied by Shandong Yousuo Chemical Co., Ltd., Heze City, Shandong Province, China. A DuPont Nafion solution (D520–1000 EW) containing 5 wt% copolymer resin was purchased from the fuel cell store. Dimethyl acetamide (DMAc) was obtained from VWR Chemicals, Budapest, Hungary. Solvents such as absolute ethanol, 1-propanol, and 1-butanol were purchased from Gilca, Labbox, and Scharlab, Barcelona, Spain, respectively. All aqueous solutions were prepared using Millipore water.

### 2.2. Synthesis of Sulfonating Montmorillonite (MMT) Nanoparticles 

The sulfonation process of montmorillonite involved five steps to reach the final S-MMT nanoparticle product: starting with protonation of the dry MMT using 2 g of Na^+^-MMT and dissolving them in 15 mL of 0.5 M H_2_SO_4_ at 30 °C for 24 h. This is followed by drying in a vacuum oven at 100 °C for 2–3 h, to obtain the protonated H^+^-MMT. The third step is to attach the thiol group (-SH) onto the surface of H^+^-MMT using (3-Mercaptopropyl) trimethoxysilane (3-MPTMS) in the presence of toluene at 110 °C for 24 h. In order to oxidize the thiol group into HSO_3_, 20 mL of 10% hydrogen peroxide was mixed with the obtained material at 80 °C for 18 h. Finally, the received powder was treated in 0.5 M H_2_SO_4_ at 30 °C for approximately 20–24 h, followed by centrifugation and drying.

### 2.3. Preparation of Membranes

**Figure 1:**
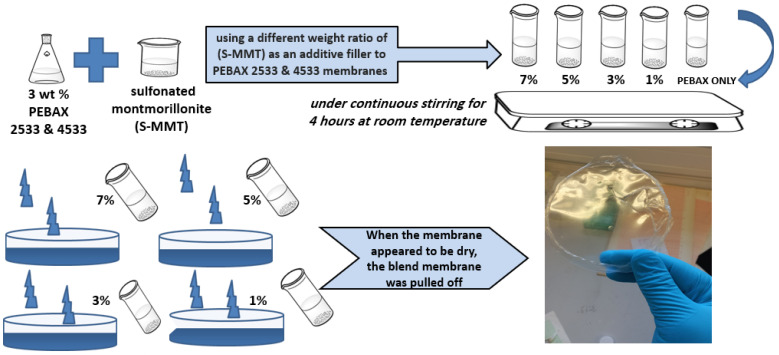
Composite membrane preparation steps.

All membranes were prepared using the casting-solution method. PEBAX 2533 was dissolved in ethanol/water (9:1) and dissolved under reflux for 2 h at 80 °C. PEBAX 4533 was dissolved in a mixture of 1-propanol and 1-butanol (3/1 (*v*/*v*)) under reflux for 3 h at 60 °C. When the polymers were totally dissolved, different amounts of S-MMT were added to the solutions under continuous stirring for 4 h at room temperature. Casting solutions (3 wt% to polymers) were poured onto glass Petri dishes and left to dry for 48 h [21]. Figure 1 illustrates the fabrication procedure of membranes. 

A perfluorosulfonic acid (PFSA) polymer, recast Nafion membrane was prepared and used in this work for comparison with our blended membranes. In the solution casting method, after evaporating the solvent from the Nafion solution, an appropriate amount of resin was redissolved in DMAC, which was then cast onto a glass Petri dish. The produced membrane was dried at 80 °C for 24 h, followed by annealing at 120 °C for 4 h. The recast membrane was then obtained after immersing it in DI water for a few minutes.

### 2.4. Membrane Characterization

In order to achieve repeatability and transparency, as well as more accurate results, we performed three repetitions from one sample during each measurement, and then determined the average of the three results.

#### 2.4.1. Water Uptake (WU) and Swelling Ratio (SR)

For the purpose of evaluating the water uptake (WU) and swelling ratio (SR) of the membranes, 2 × 2 cm^2^ square samples of the membranes were immersed in deionized (DI) water for 24 h. Subsequently, the surface water was removed using tissue paper and wet mass and wet dimensions of the membranes were measured. The samples were then dried overnight in a vacuum oven at 50 °C. Finally, the mass and dimensions were determined again. The calculations for the water uptake and swelling ratio were derived from Equations (1) and (2), respectively.

(1) $$WU(\%)=\frac{W_{wet}-W_{dry}}{W_{dry}}\;\times \;100$$

where W_wet_ and W_dry_ represent the weight of the samples in the wet and dry state, respectively.

(2) $$SR(\%)=\frac{A_{wet}-A_{dry}}{A_{dry}}\;\times \;100$$

The terms “A_dry_” and “A_wet_” refer to the dry and wet areas of the membrane, respectively.

#### 2.4.2. Ion Exchange Capacity (IEC)

The measurement of the ion exchange capacity (IEC in millimol equivalents per gram, meq/g) of the prepared membranes was performed using acid–base titration. First, the samples were placed in 20 mL of a 1 M NaCl solution for 24 h to replace the H^+^ ions with Na^+^ ions. The solution containing the released H^+^ ions was then titrated with a 0.1 M NaOH solution. IEC was calculated from Equation (3).

(3) $$IEC(meq/g)=\frac{C_{NaOH}\times V_{NaOH}}{W_{dry}}$$

where C_NaOH_, V_NaOH_, and W_dry_ are the concentration of the NaOH titration solution, the volume of NaOH consumed, and the weight of the dried membranes, respectively. It should be emphasized that neither the new PEBAX-based membranes nor the recast Nafion were pre-protonated.

#### 2.4.3. TG-MS Analysis (TGA)

The thermal stability of the membranes was investigated with a TG-MS analysis using a Perkin Elmer TGS-2 thermobalance equipped with a modified furnace and a temperature controller (Eurotherm, Worthing, UK). The thermobalance was coupled to a Pfeiffer HiQuad (Dresden, Germany) quadrupole mass spectrometer. Approximately 4.5 mg of samples was weighed into a platinum sample holder under an argon atmosphere at a flow rate of 140 mL/min. The heating rate of 20 °C/min was applied, from the ambient temperature up to 900 °C. The effluent gas mixture was introduced into the mass spectrometer through a heated capillary line. Ionization energy of 70 eV was used. The measured ion intensities were normalized to the intensity of the ^38^Ar isotope of the carrier gas and to the initial sample amount.

#### 2.4.4. Morphological Analysis by Using Scanning Electron Microscopy (SEM)

Scanning Electron Microscopy (SEM) of the composite membranes was performed to examine the surface morphology by using ZEISS EVO 40XVP SEM (Oberkochen, Baden-Württemberg, Germany). It is a variable pressure scanning electron microscope (VP-SEM). The microscope is suitable for a general-purpose microstructural analysis at high vacuuming. 

#### 2.4.5. Fourier Transform Infrared Spectroscopy (FTIR)

The interaction between the S-MMT and PEBAX polymer chains was investigated using Fourier Transform Infrared Spectroscopy (FTIR) in the attenuated total reflectance (ATR) mode. The FTIR analysis was conducted using Tensor II equipment manufactured by Bruker (Ettlingen, Germany), covering a spectral range of 400–4000 cm^−1^. The Fourier Transform Infrared (FTIR) spectra of each membrane were obtained by averaging sixteen scans.

#### 2.4.6. X-ray Diffraction (XRD)

The X-ray diffraction patterns of PEBAX2533 and PEBAX4533 membranes, including varying ratios of S-MMT nanofiller, were obtained using a Philips model PW 3710-based PW1050 Bragg-Brentano Para focusing goniometer, Malvern Panalytical Ltd., Prague, Czech Republic. The X-ray source used Cu Kα radiation with a wavelength (λ) of 0.15418 nm, and the diffraction angles (2Θ) were measured in the range of 4°–75°. The lattice parameters were determined through full profile fitting, specifically the Pawley fitting method.

#### 2.4.7. Mechanical Stability

The mechanical characteristics of all membranes were determined using universal testing equipment (Zwick Z005 GmbH & Co., KG, Ulm, Germany). The membranes had dimensions of 75 mm × 10 mm and were tested at a speed of 20 mm/min with an initial grip distance of 35 mm.

#### 2.4.8. Chemical Stability 

To determine the chemical stability of the synthesized composite membranes, a hot Fenton’s reagent (3 wt% H_2_O_2_ and 4 ppm of Fe_2_O_4_) was used. The different (2 × 2 cm^2^) samples were cut and labelled, and the synthesized composite membrane was dried in an oven at 60 °C for 24 h. All the samples were then weighed with the help of a sensitive balance. The sample was immersed in Fenton’s solution at 80 °C for 24 h, and after the samples were removed from the solution, they were dried and weighed to obtain the percent weight loss [22].

#### 2.4.9. Ion Conductivity

The ion conductivity of the membrane was determined using potentiostatic electrochemical impedance spectroscopy (PEIS) in the frequency range of 1 Hz to 1 MHz. An oscillating voltage with an amplitude of 10 mV was applied, and the resistance of the membranes was measured using a two-compartment cell with an exposed area of 1 cm^2^. The electrical resistance of the cell was determined by using a two-probe impedance technique, both with and without the presence of the membrane, at room temperature conditions. It should be emphasized that neither the new PEBAX-based membranes nor the recast Nafion were pre-protonated. The ion conductivity of the membranes was calculated with the help of Equation (4) [23].

(4) $$\sigma =\frac{L}{R\times A}$$

In the given context, σ (mS/cm) represents the ion conductivity, L (cm) denotes the thickness of the membrane, R (Ω) is the resistance of the membrane, and A (cm^2^) is the exposed surface area of the membrane.

## 3. Results and Discussion

**Figure 2:**
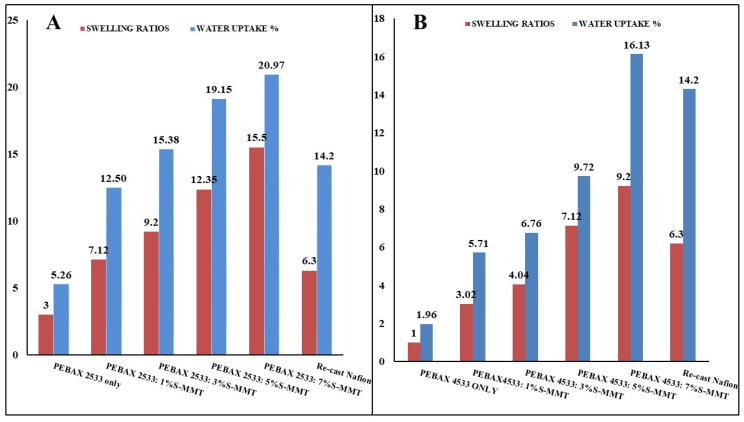
Water uptake and swelling ratio for PEBAX 2533 and for PEBAX 4533 (**A**,**B**) at different S-MMT content.

Figure 2 illustrates both the water uptake and the swelling ratios of the membranes when they were exposed to DI water at room temperature. Both the water uptake and the swelling ratio increased with the increasing sulfonated montmorillonite (S-MMT) content because S-MMT might enhance hydrophilicity due to the presence of the sulfonyl group. This property facilitates the absorption of water molecules, thereby promoting increased water uptake of the membrane. In addition, the capillary effect can be facilitated by the porous nature of MMT, which allows water to be attracted and move into the interlayer spaces. When the montmorillonite (MMT) is dispersed in the polymer matrix, water molecules are able to adsorb between the layers of MMT. The increased interlayer space facilitates the accommodation of a greater amount of water molecules, which is clearly visible at 7% S-MMT content for both PEBAX2533 and PEBAX4533. 

PEBAX2533 with 7% S-MMT achieves a greater water uptake and swelling degree than PEBAX4533 with the same ratio of S-MMT, which is reported to be beneficial in terms of protection against membrane failure phenomena [24]. Comparing the results of the water absorption and swelling degree of recast Nafion, it was clearly indicated that our blended membranes gave better results than recast Nafion [10]. However, too high a swelling ratio can have a detrimental effect on fuel cell performance. In general, a good balance between the water uptake and swelling ratio should be achieved. Fine-tuning the filler content and thus the optimal ratio between the water uptake and swelling ratio is the subject of a next article, where membrane–electrode assemblies under fuel cell conditions will be investigated.

**Figure 3:**
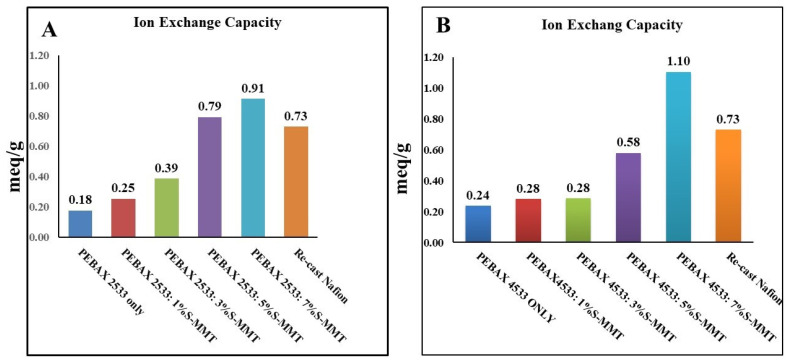
Ion exchange capacity of PEBAX 2533 and PEBAX 4533 (**A**,**B**) with different S-MMT contents.

The ion exchange capacity of a membrane reveals the concentration of cations that can be exchanged with protons. Hence, this correlates strongly with proton conductivity, which is one of the most important aspects to consider when selecting membranes for fuel cell applications. The IEC test results for the blended membranes can be seen in Figure 3A,B. From the results, it can be observed that increasing the S-MMT content leads to an increase in IEC. For PEBAX2533, the highest IEC is achieved by 7% S-MMT with the value of 0.9 meq/g compared to 0.2 meq/g for the pristine PEBAX2533 membrane. Meanwhile, for PEBAX4533, the highest IEC is achieved by the same S-MMT content with the value of 1.10 meq/g compared to 0.24 meq/g for the pristine PEBAX4533 membrane. Due to the dangling terminal hydroxyl or amide groups, the PEBAX membrane has an internal ion exchange capacity. However, the addition of montmorillonite further increases this characteristic. The higher IEC can be attributed to the presence of sulfonic acid groups on the surface of sulfonated montmorillonite, which provide additional sites for ion exchange. The surface of natural montmorillonite is ca. 60 m^2^/g [25], which is already large enough for a high concentration of functional groups on the surface. However, under mild conditions, the clay can easily be exfoliated into 1.6-nm-thick nanoplatelets [26]. The surface area of two-dimensional montmorillonite nanosheets can reach ca. 470 m^2^/g. Moreover, it has a large adsorption capacity too [25]. It is expected that during the 4 hours of mixing in the alcoholic solutions used to dissolve the PEBAX polymers, the montmorillonites are largely exfoliated and the nanosheets are uniformly distributed in the polymer matrix. Eventually, the resulting composite represents the continuous spatial presence of ion exchange sites. Consequently, the use of S-MMT may lead to enhanced ion mobility, which may facilitate ion transport across the membrane. It is shown in the related art that membranes with a high water uptake capacity also had a high ion exchange capacity. Figure 3 illustrates a marginal difference only in ion exchange capacity between PEBAX2533 and PEBAX4533 with a 7% S-MMT. The higher polyether content (80%) of PEBAX2533 results in a slightly lower ion exchange capacity compared to PEBAX4533, which contains approximately 50% polyether. It is clearly evidenced that the synthesized membranes performed more efficiently than the recast Nafion when the ion exchange capacity test was compared [10]. In other words, it can be assumed that good results can be achieved by using the PEBAX2533 and PEBAX4533 membranes synthesized with S-MMT filler in a fuel cell.

**Figure 4:**
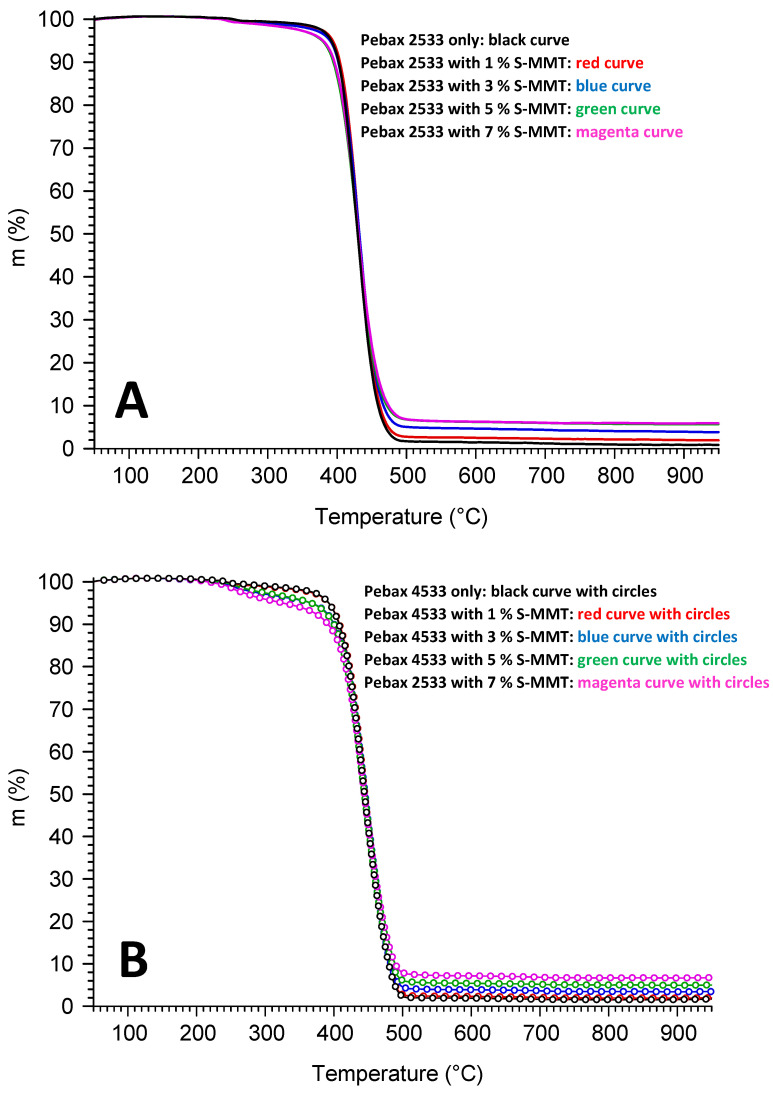
The thermal stability of PEBAX 2533 and PEBAX 4533 (**A**,**B**) with different S-MMT content.

The thermal stability of PEBAX2533 and PEBAX4533 membranes with different S-MMT contents was investigated through a thermogravimetric analysis under Ar. The TGA analyses are shown in Figure 4. As can be seen in the figure, the degradation of all membranes starts above 300 °C, which coincides well with the literature data that the thermal degradation range for the pure PEBAX membrane is between 350 and 480 °C [13,21]. The decomposition of the polyamide segment results in the production of cyclic monomers, α-olefins, and dimers [20]. Thermal stability of the blended membranes has a significant correlation with filler concentration. The experimental results indicate a drop in the onset decomposition temperature with increasing filler concentration. It is clear that the S-MMT blended membranes are less stable than the pure forms of PEBAX 2533 and PEBAX 4533 membranes [21]. However, similar to the pristine polymers, the blended membranes also degrade in one step, which suggests that the incorporation of the S-MMT filler did not affect the mechanism of thermal degradation of the membranes.

Table 1 shows the amount of carbonaceous residue (char) obtained after decomposition of parent and blended membranes with various S-MMT contents at 950 °C. There is only a slight difference in the thermal stability of PEBAX 2533 and PEBAX 4533, which may be attributed to the difference between the ratios of rigid and flexible components. The hard block (polyamide) in PEBAX 4533 is present in a greater proportion, which results in improved thermal resistance. In contrast, the existence of a large proportion of PTMO in PEBAX 2533 enhances the mobility of polymer chains and consequently accelerates the thermal degradation of the polymer [21]. 

**Figure 5:**
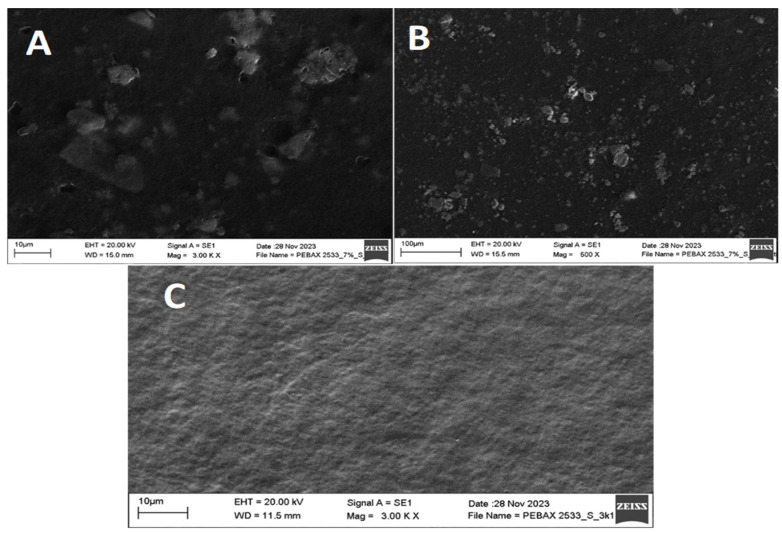
SEM images of PEBAX 2533 with 7% S-MMT filler (**A**,**B**) and without S-MMT filler (**C**).

**Figure 6:**
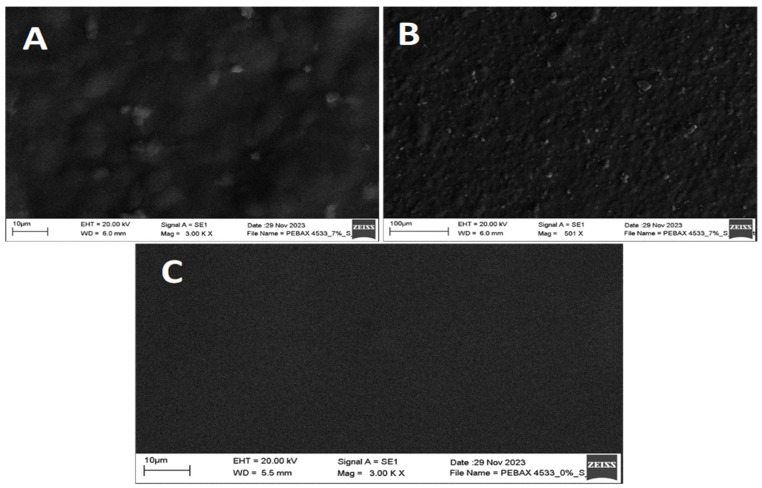
SEM images of PEBAX 4533 with 7% S-MMT filler (**A**,**B**) and without S-MMT filler (**C**).

Surface morphology of samples has been investigated using Scanning Electron Microscopy (SEM). Figure 5 and Figure 6 show SEM micrographs of PEBAX 2533- and PEBAX 4533-based blended membranes containing S-MMT filler. The surface morphology indicates the compatibility of the organic polymer and the inorganic S-MMT particles, which is of great importance in determining the thermal, mechanical, and optical properties of the membranes. It is evident from the images in Figure 5 and Figure 6 that the dispersion of 7% for the S-MMT filler in the PEBAX matrix of all the membranes is acceptably uniform and well distributed, which confirms that it allows good distribution of the particles. Overall, the synthesized composite membranes have a solid, homogeneous, smooth, and defect-free surface.

**Figure 7:**
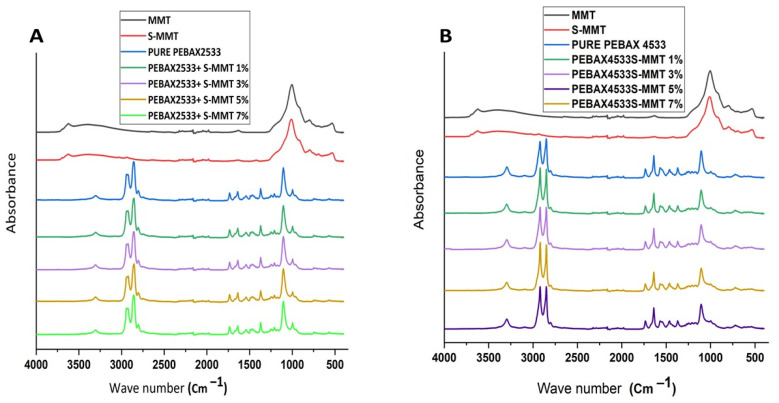
FTIR spectra of PEBAX 2533 and PEBAX 4533 (**A**,**B**) with different S-MMT content.

FT-IR spectroscopy was used to identify the functional groups present and to determine the nature of interaction between the PEBAX 2533 and PEBAX 4533 matrices and the S-MMT filler. The FT-IR spectra are shown in Figure 7. It is clear that for pure PEBAX2533, the bands in the spectrum correspond to different vibrations of the various functional groups present in the polyether and polyamide blocks of the membrane. Specifically, they can be attributed to the stretching vibrations of (C-H), (C-O), (C=NH), and H-N-C=O of free amide, (C-H group), and (N-H) groups [12,21]. Figure 7A,B also show the spectra of the parent and sulfonated montmorillonite (MMT and S-MMT). The peaks at 521 cm^−1^ and 1033 cm^−1^ correspond to the Al–O bending and Si–O stretching vibrations, respectively, which are typical peaks of MMT. Additionally, the peaks observed at 3620 cm^−1^ and 3435 cm^−1^ are attributed to the stretching vibrations of the Al–O–H bond and H–O–H bond, respectively, which provide information about the water absorbed [20]. The presence of sulfonic acid groups in S-MMT can be confirmed using the appearance of characteristic peaks at 1300 cm^−1^ corresponding to (S=O) stretching vibrations. 

**Figure 8:**
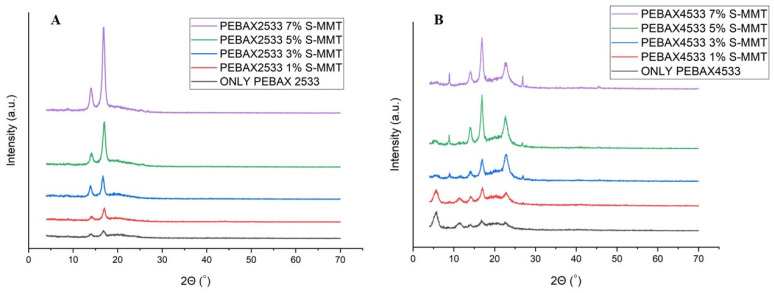
The X-ray diffraction (XRD) patterns of PEBAX 2533 and PEBAX 4533 (**A**,**B**).

The X-ray diffraction (XRD) patterns of the membranes are shown in Figure 8A,B. The semicrystalline character of PEBAX-type copolymers is attributed to the presence of both amorphous PTMO and crystalline PA12 segments [21]. The observed five peaks at 2θ = 5.7°, 11.1°, 14.2°, 17.9°, and 22.3° are in agreement with the peak positions assigned to the PA12 component of the parent PEBAX 4533 polymer. In the case of pure PEBAX 2533, two distinct peaks obtained at 13.7° and 18.1° are also attributed to PA12 [13]. The observed deviance in the peak intensities between two pristine PEBAXs can be attributed to the difference in the relative ratio of rigid and flexible segments. According to the spectra shown in Figure 8A,B, with the addition of nanofiller to the PEBAX 2533 matrix, the intensity of the peaks at 13.7° and 18.1° gradually increased with the increase in the amount of S-MMT. This rise in intensity suggests an enhancement in membrane crystallinity. As a nucleating agent, S-MMT can promote formation and growth of nuclei of polymer segments. For the PEBAX4533 series, the two peaks (at 5.7° and 11.1°) seen in the parent and 1%-S-MMT-loaded PEBAX4533 were absent in the XRD patterns of blends with 3%, 5%, and 7% nanofiller content. Simultaneously, with the increase in filler content, new peaks appeared at 9.5° and 27.4°, with a gradual increase in intensity. The strong compatibility between S-MMT and PEBAX 2533, as well as PEBAX 4533 matrices, improved the crystallization process of PEBAX.

The mechanical strength of a membrane is an important feature. According to research, the incorporation of nanosized inorganic fillers into hybrid membranes has been identified to be an effective and simple approach to improve the mechanical properties of polymers [21]. In order to investigate the effect of S-MMT nanofiller on the mechanical properties of PEBAX2533 and PEBAX4533 membranes, tensile tests were performed. The results are shown in Figure 7A for PEBAX2533. The composite membrane consisting of PEBAX2533 blended with 3% S-MMT as nanofiller exhibited the lowest strength, with a maximum stress value of 3.7 MPa. In comparison, the presence of the S-MMT nanofiller at 1% S-MMT and 5% S-MMT resulted in an increase in membrane strength (5.7 and 4.5 MPa for PEBAX2533 with the S-MMT nanofiller, respectively), but with a high elongation at break ratio. However, the addition of a greater percentage of S-MMT (specifically, 7% S-MMT) resulted in a significant reduction in the elongation at break ratio, specifically seen at a maximum tensile stress of 4.5 MPa. The observed phenomenon is probably attributed to the large PA12 crystallites within the PEBAX polymer chains, as evidenced through the XRD analysis. Nevertheless, the parent PEBAX material exhibited a higher tensile strength and greater elongation at the breaking point in comparison to all the membranes prepared by blending varying proportions of S-MMT. The good mechanical properties are most probably due to an ideal combination of features of polyamide and polyether blocks. The added value of S-MMT is due to its impact on the IEC, ion conductivity, and swelling ratio while not significantly degrading the mechanical properties. The membrane with 5% S-MMT behaves like the parent PEBAX2533 in terms of mechanical strength. 

**Figure 9:**
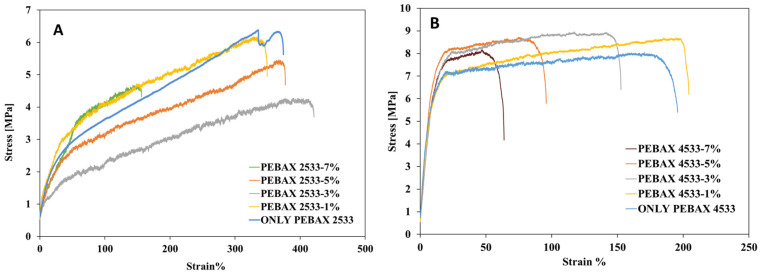
Mechanical stability of PEBAX 2533- and PEBAX 4533-based blended membranes (**A**,**B**) with different S-MMT content.

Results of mechanical stability in the case of PEBAX4533 series are shown in Figure 9B. It is clearly seen that increasing the nanofiller ratio (S-MMT) leads to an increase in the tensile strength in the blended membrane composed of PEBAX4533 and S-MMT. The parent PEBAX4533 showed the minimum tensile strength, reaching a maximum tensile stress of 7.5 MPa in addition to a strain ratio of around 180%. When comparing the effects of incorporating S-MMT nanofiller at different concentrations (1, 3, and 5%) in the membrane, it is observed that the addition of 1% S-MMT resulted in an increase in tensile stress force of 8.2 MPa, corresponding to a strain ratio of approximately 210%. The incorporation of 3% S-MMT led to a higher increase in membrane strength, reaching 8.7 MPa with an elongation at break of approximately 150%. The use of 5% S-MMT nanofiller in PEBAX4533 resulted in a membrane strength of 8.5 MPa with an elongation at break of approximately 100%. The inclusion of a higher amount of S-MMT (specifically, 7% S-MMT) resulted in a significant reduction in the elongation at break ratio (75%) and a maximum stress of 8 MPa. Nevertheless, the mechanical stability of the blended membranes composed of PEBAX 4533 and varying amounts of S-MMT exhibited favorable properties when compared to PEBAX 2533 series. The incorporation of PA12 and PTMO segments inside the PEBAX polymer matrix produces a material with exceptional versatility, demonstrating favorable attributes such as flexibility, toughness, and superior resistance to chemical degradation. The addition of S-MMT can fine-tune these properties, for example, through its effect on the crystallinity of the PA12 segment. 

**Figure 10:**
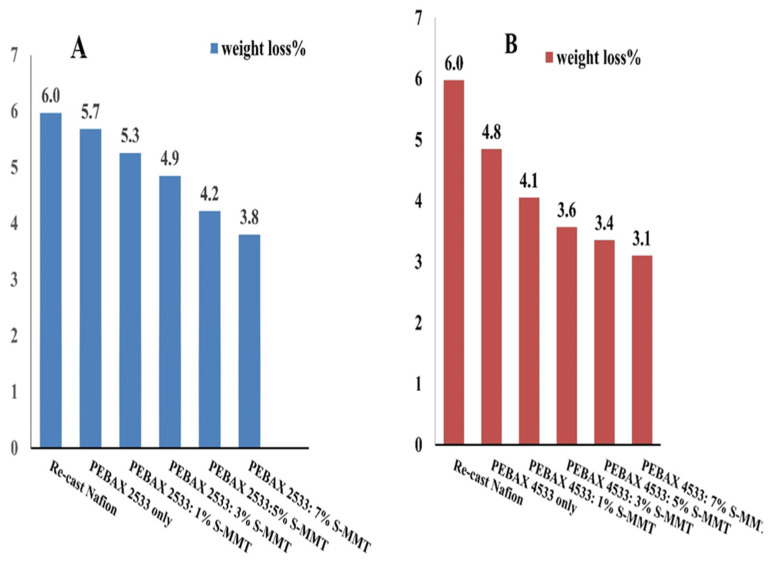
Chemical stability based on Fenton’s test of PEBAX 2533- and PEBAX 4533-based blended membranes (**A**,**B**) with different S-MMT content.

Fenton’s reagent test is a chemical stability test that is broadly used to measure the resistance of a PEM against the attack of radical species (OH· and OOH·) that get generated at the two electrodes of a PEMFC [22]. Figure 10 shows the percentage weight loss of the synthesized membranes under Fenton’s reagent test with immersion of all the samples for 24 h at 80 °C. The weight loss of the synthesized composite membranes is due to the attack of radical species on the PEBAX matrix. A significant difference in the weight loss between the membranes is observed as a function of S-MMT content. The total weight loss decreased with increasing filler content, as S-MMT may exhibit free radical scavenging properties or act as an antioxidant, reducing the effect of hydroxyl radicals generated during the Fenton reaction. This behavior could help mitigate oxidative damage to the polymer matrix. In addition, blended membranes based on PEBAX 4533 have been shown to be less sensitive to Fenton’s reagent than the PEBAX 2533 series. This phenomenon can be explained by the fact that PEBAX 4533 contains a higher proportion of the polyamide (PA12) hard segment, approximately 50%, than PEBAX 2533, which only contains approximately 20%. [18]. The presence of PA12 inside the PEBAX 4533 polymer matrix results in high toughness and excellent resistance to chemical degradation. As a result, the synthesized membranes with high S-MMT content were able to maintain structural integrity without punctures or breakage. It was evident that the chemical stability of the synthesized membranes was better than that of recast Nafion. The synthesized PEBAX2533 and PEBAX4533 membranes with S-MMT filler are expected to perform well in a fuel cell.

Potentiostatic electrochemical impedance spectroscopy (PEIS) was used to measure the membrane’s ion conductivity. We wanted to investigate the impact of S-MMT nanofiller on the conductivity of PEBAX2533 and PEBAX4533 membranes. The results are shown in Table 2. The ion conductivity of the membranes showed an improvement by increasing the S-MMT content. The ion conductivity results correlate with the ion exchange capacity results of the PEBAX2533 and PEBAX4533 blend membranes. The highest ion conductivity at 25 °C was obtained when using 7% S-MMT, which is 2 mS cm^−1^ and 1.6 mS cm^−1^ for PEBAX2533 and PEBAX4533, respectively. The parent PEBAX2533 and PEBAX4533 membranes had the lowest values of 1 and 0.9 mS cm^−1^, respectively. The observed phenomenon can be attributed to high specific surface area of sulfonated montmorillonite owing to its layered structure. As a result, there is a high concentration of sulfonic acid groups exposed on the surface. Hence, when blended with PEBAX, it significantly enhances the surface concentration sites available for cation transport. This has the possibility to improve the total ion conductivity of the membrane [19,24]. According to Table 2, lower ion conductivity values were obtained for the PEBAX4533 series than for PEBAX2533 ones, despite the fact that PEBAX4533-based membranes have a slightly higher ion exchange capacity as emerging from Figure 3B. 

## 4. Conclusions

Novel, PEBAX-based ion exchange membranes containing sulfonated montmorillonite (S-MMT) as filler have been manufactured and investigated. PEBAX block copolymers with varying polyamide and polyether segment ratios (PEBAX2533 and PEBAX4533) have been applied. Membranes were synthesized using the solution casting method.

With an increase in the S-MMT content, the majority of the desirable properties have improved for both types of PEBAX (2533 and 4533), including IEC, ion conductivity, mechanical stability, water uptake, and thermal stability. However, for the PEBAX2533 series, compared to the parent membrane, the mechanical properties slightly deteriorated with increasing S-MMT content; all the membranes demonstrated acceptable mechanical stability. By increasing the S-MMT content from 0% to 7% in the PEBAX matrix, IEC shows a significant increase from 0.24 to 1.10 meq/g for PEBAX 4533 and from 0.2 to 0.9 meq/g for PEBAX 2533. In terms of ion conductivity, for membranes based on PEBAX 2533, it increased from 1 to 2 mS cm^−1^, while for PEBAX 4533, the ion conductivity grew from 0.9 to 1.6 mS cm^−1^. A prerequisite for high ion exchange capacity and ion conductivity is the appropriate exfoliation of S-MMT and even distribution of montmorillonite nanosheets in the polymer matrix. The 4-hour-long agitation of S-MMT in the alcoholic solution of the PEBAX polymer proved to be a suitable method for creating a homogeneous composite matrix. Highly exfoliated S-MMT can have an extremely high surface area with a high concentration of surface sulfonic acid groups that provide ion exchange sites on the surface. The spatially uninterrupted presence of S-MMT in the polymer matrix must contribute to ionic conductivity. 

The above results encourage us to further modify our membranes and test them under in situ fuel cell conditions. 

## Figures and Tables

**Table 1:** Carbonaceous residue (char) obtained at a temperature of 950 °C for various ratios of S-MMT in blend membranes.

Sample	Char (%)
Pebax 2533 only	0.84
Pebax 2533 with 1% S-MMT	1.95
Pebax 2533 with 3% S-MMT	3.82
Pebax 2533 with 5% S-MMT	5.67
Pebax 2533 with 7% S-MMT	5.84
Pebax 4533 only	1.52
Pebax 4533 with 1% S-MMT	1.89
Pebax 4533 with 3% S-MMT	3.40
Pebax 4533 with 5% S-MMT	4.92
Pebax 2533 with 7% S-MMT	6.64

**Table 2:** Ion conductivity of blended membranes with various amounts of S-MMT at 25 °C.

Type of Synthesized Membrane	Thickness of the Membrane (µm)	Ion Conductivity (mS/cm) at 25 °C	Type of Synthesized Membrane	Thickness of the Membrane (µm)	Ion Conductivity (mS/cm) at 25 °C
Only Pebax 2533	46.2	1	Only Pebax 4533	45	0.9
Pebax 2533 with 1% S-MMT	46.8	1.3	Pebax 4533 with 1% S-MMT	45.6	1.4
Pebax 2533 with 3% S-MMT	47.6	1.7	Pebax 4533 with 3% S-MMT	46.3	1.5
Pebax 2533 with 5% S-MMT	47.8	1.8	Pebax 4533 with 5% S-MMT	46.7	1.5
Pebax 2533 with 7% S-MMT	48.3	2	Pebax 4533 with 7% S-MMT	47.4	1.6
Recast Nafion [10]	27.8	4.8	Recast Nafion [10]	27.8	4.8

## Data Availability

The data presented in this study are available on request from the corresponding author. The data are not publicly available due to [their unstandardizable complexity].
